# Intrinsically disordered proteins: modes of binding with emphasis on disordered domains

**DOI:** 10.1098/rsob.210222

**Published:** 2021-10-06

**Authors:** Owen Michael Morris, James Hilary Torpey, Rivka Leah Isaacson

**Affiliations:** Department of Chemistry, Faculty of Natural, Mathematical and Engineering Sciences, King's College London, Britannia House, 7 Trinity Street, London SE1 1DB, UK

**Keywords:** intrinsic disorder, fuzzy binding, protein folding

## Abstract

Our notions of protein function have long been determined by the protein structure–function paradigm. However, the idea that protein function is dictated by a prerequisite complementarity of shapes at the binding interface is becoming increasingly challenged. Interactions involving intrinsically disordered proteins (IDPs) have indicated a significant degree of disorder present in the bound state, ranging from static disorder to complete disorder, termed ‘random fuzziness’. This review assesses the anatomy of an IDP and relates how its intrinsic properties permit promiscuity and allow for the various modes of interaction. Furthermore, a mechanistic overview of the types of disordered domains is detailed, while also relating to a recent example and the kinetic and thermodynamic principles governing its formation.

## Graphical abstract

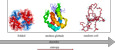

## 1. Introduction

The historic protein structure–function paradigm dictates that in order for a protein to function, it must adopt a specific three-dimensional structure. This three-dimensional structure is determined by the primary sequence of the polypeptide chain and its intrinsic properties. This paradigm implies that the function of a protein is determined by its structure and by extension from its sequence [1]. This led to the notion that interactions involving proteins are determined by the complementary shapes at the binding interface and the resulting non-covalent forces between biomolecules [2].

Decades of progress in structural biology have shaped the protein structure–function paradigm, where many thousands of protein structures and complexes have been studied comprehensively at an atomic level [3]. However, the idea that a well-defined structure is a pre-requisite for protein function is becoming increasingly challenged by recent observations of intrinsically disordered proteins (IDPs) exerting their function in a complex that has an absence of any detectable secondary structure [2].

Over the past three decades, it has become apparent that a large percentage of any organism's proteome consists of proteins or protein regions that lack any form of well-defined secondary structure [4]. These proteins are said to be intrinsically disordered. Their prevalence was suggested as early as the late 1990s when neural networks flagged more than 15 000 members of the Swiss Protein Database as likely to contain disordered regions of more than 40 residues [5]. In this same time period, several key publications emerged describing the link between the IDPs tau and NACP (now known as α-synuclein) to neurodegenerative disease, which helped fuel interest in IDPs among the structural biology community [6–9]. An IDP is defined as a protein that lacks a unique fold, either entirely or in parts when isolated in solution [10]. IDPs exist as a dynamic ensemble of rapidly interconverting conformers in equilibrium [10–12]. Despite the lack of secondary structure, IDPs exhibit functionality. A functional protein that exists as a multitude of conformations allows for a single IDP to interact promiscuously with many different partners. Studies have shown that proteins containing intrinsically disordered regions (IDRs) hold central roles in protein interaction networks, specifically acting as hub proteins within the nucleus and enabling molecular communication via protein–protein interactions (PPIs) [13–15].

The modes of interaction between an IDP and its target are vast. The majority of IDPs adopt three-dimensional structures once bound to their targets in a phenomenon known as ‘folding-upon-binding’ [15]. The conformational plasticity of an individual IDP allows for a range of secondary structures to be induced in the bound state of its promiscuous interactions; an example is the tumour suppressor protein, p53, which has over 500 interaction partners on the STRING database and adopts an array of structures in complex [10]. However, the highly dynamic nature of IDPs allows for PPIs where one or both partners are able to retain a significant degree of their structural heterogeneity [2,10]. The degree of disorder in the bound state may be thought of as a continuum in flux between enthalpic and entropic contributions, rather than mutually exclusive modes of interaction. While basic secondary structure may be induced upon the binding of an IDP to a folded partner, the enthalpic contributions of binding may not be sufficient to pay the entropic penalty, and the protein will exhibit both ordered and disordered transitions in the bound state.

This review assesses the types of ordered and disordered interaction domains in which disorder may be found. For each complex, a description will be offered on how the anatomy of an IDP enables the formation of these interaction domains, along with providing the kinetic and thermodynamic principles governing their formation.

## 2. Anatomy of an intrinsically disordered protein

**Figure 1.**
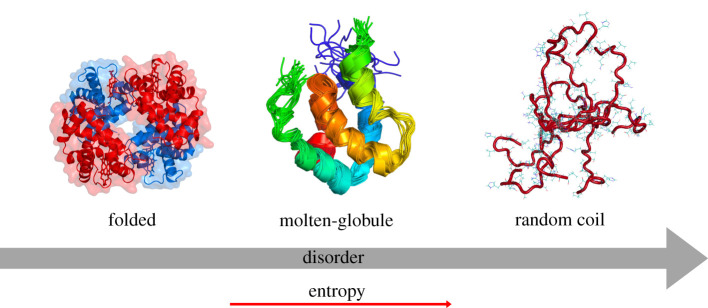
The protein folding continuum. Spectrum of disorder characterized by the entropy value. Proteins are identified as having a well-defined three-dimensional structure, a molten-globule structure consisting of folded ordered and disordered regions or a completely disordered random coil. Depiction illustrates folded regions being less dynamic than coils near the N- and C-termini. Random coils have no inherent three-dimensional structure and the vast number of polar and charged amino acid residues is shown in stick format. Folded structure depicts PDB 1A3N, molten-globule structure depicts 6ES6 and random coil depicts 1L1 K [16–18].

Most proteins consist of a densely packed hydrophobic core and exist as a minimum free energy native fold. Towards the N- and C-termini, regions of the polypeptide may become more dynamic, as residues are less constrained and interact more freely with the surrounding aqueous environment. Conversely, there are proteins that completely lack a hydrophobic core and exist as a highly dynamic ensemble of conformations. The protein folding continuum compares the entropy values of a given protein relative to a well-defined structure and an IDP (figure 1) [10]. A number of proteins contain both ordered and disordered domains. These may exist as molten-globules or possess random coil domains. Therefore, it is essential that we differentiate between proteins that completely lack any form of secondary structure and exist in an equilibrium of rapidly interconverting conformers, IDPs, and proteins that consist of both ordered and disordered domains, which are said to contain IDRs.

### 2.1. Identifying IDP(R)s by their characteristic amino acid sequence

Intrinsic disorder in proteins is attributed to their scarce proportions of hydrophobic and aromatic amino acids [19]. Protein folding is driven by the hydrophobicity between the surrounding aqueous cellular environment and the hydrophobic amino acid residues. The phobicity between the cytosol and hydrophobic residues results in the emergence of a hydrophobic core, thereby optimizing the intramolecular interactions, and stabilizing a protein fold. Due to the inherent lack of bulky hydrophobic and aromatic amino acid residues within IDPs, a hydrophobic core cannot be established to drive the formation of a well-defined, three-dimensional protein fold [20]. IDP(R)s are rich in charged, hydrophilic amino acid residues (Lys, Asp). Large numbers of opposingly charged amino acid residues in a continuous stretch will destabilize the formation of any compact state. Moreover, the copious number of polar and charged residues present under physiological conditions, combined with the lack of intrinsic affinity, results in there being far fewer intramolecular hydrogen bonds [4]. Instead, IDP(R)s interact freely via hydrogen bonding with the surrounding aqueous solvent. Simpler amino acids, such as serine, proline, glycine and glutamine, are abundant in IDP(R)s. Proline is unable to participate in regular hydrogen bonding due to the lack of a free backbone amide. The relative rarity of intramolecular hydrogen bonding prevents the formation of a compact three-dimensional state. Meanwhile, these abundant simple amino acids, especially glycine, possess a great deal of conformational freedom, resulting from a minimized steric hindrance involved with its side chain. The total lack of intrinsic affinity, paucity of hydrophobic residues and high proportion of flexible amino acids results in the IDP(R) possessing a great deal of conformational flexibility, characterized by a large entropic value. Furthermore, the plasticity of the IDP(R) allows for promiscuous interactions with many partners, adopting different folds for different partners or lacking a fold altogether when in the bound state [21]. To further serve their function, IDPs have a large surface area per amino acid residue, provided by the lack of intramolecular interactions. This allows for greater exposure to single or multiple linear motifs [4]. This property is heavily exploited with post-translational modifications (PTMs) of IDP(R)s. PTMs may modify activity by altering relative hydrophobicity and local charge density in processes such as phosphorylation of nuclear proteins [4].

### 2.2. Modules of IDP(R)s

The primary sequences of IDPs may be grouped into three modules: molecular recognition features (MoRFs), short linear motifs (SLiMs) and low-complexity regions (LCRs) [13,22,23].

Molecular recognition is a process where biological entities specifically interact with each other or smaller molecules to form a complex. MoRFs are short motifs, 10–70 amino acid residues in length, that remain intrinsically unstructured and exist to achieve molecular recognition [24]. MoRFs promote specific PPIs and undergo disordered-to-ordered transitions upon binding to their target [22,23]. The unbound form of an MoRF is typically biased towards the conformations which it may adopt in complex [22].

Cumberworth *et al.* [13] show that MoRFs can be divided into four sub-types according to their three-dimensional fold which becomes induced upon binding to a target: α-MoRFs, which form α-helices; β-MoRFs, which form β-strands; ι-MoRFs, which form irregular structures; and finally, complex-MoRFs, which form a mixture of secondary structures [13,25]. p53 exemplifies the activity of the sub-types of MoRFs. The first α-MoRF, located within close proximity of the N-terminus, interacts with the protein MDM2, resulting in the α-helical structure being induced [13]. This MoRF also demonstrates a wide range of promiscuous interactions, with over 40 known partners [1]. The structures resulting from MoRFs binding to their target can be highly specific to an interface and can occur with a great deal of affinity, defying the protein structure–function paradigm [13]. The process of binding is coupled with a significant loss in entropy of the IDR. However, the gain in enthalpy coupled with folding is sufficient to pay the entropic penalty of binding and make the overall process feasible. This change in enthalpy is dependent upon the size of the disordered interface and, due to the increased surface area per amino acid residue, IDRs allow for larger interfaces and thus larger gains in enthalpy, making folding-upon-binding a favourable process [26].

SLiMs are short conserved sequences, usually no longer than 10 amino acid residues long. These are responsible for mediating PPIs involved in cellular signalling and are located within disordered regions that form interfaces with partner proteins [27]. SLiMs contrast MoRFs as upon interaction, the resulting domain between a SLiM and its target may be ordered or disordered. Therefore, SLiMs are considered separate to MoRFs and are categorized based upon their sequence rather than structure. SLiMs may be divided into two major families, each with three sub-types. The first family comprises enzyme binding and modification motifs [1]. SLiMs in this family are responsible for mediating post-translational processing by acting as sites for proteolytic cleavage. SLiMs are also sites of PTMs, and many enzymes specifically seek their primary sequences. The second major family comprises complex formation motifs, which function in protein scaffolding and increasing the avidity of IDP interactions.

LCRs are sequences that encode a low level of sequence information, as quantified by Shannon's entropy [28]. The amino acid composition of LCRs is highly repetitive and composed of a select few amino acids. Those regions identified to contain LCRs have been shown to exhibit higher levels of binding promiscuity [29]. The most extensively studied LCR is polyglutamine, which holds a role in the aggregation of proteins, forming an amyloid state associated with numerous neurodegenerative diseases [30].

## 3. Interactions of IDPs

### 3.1. Ability of IDP(R)s to interact promiscuously

The intrinsic properties of an IDP(R)'s primary sequence confer great conformational malleability and make IDP(R)s ideally suited to recognizing multiple partners [31]. Analysis of PPI networks has revealed that hub proteins are enriched in disorder and that proteins increase their number of binding partners by having SLiMs distributed throughout their disordered segments [13]. p53 serves as an example and a reason for its existence may lie within its plasticity, enabling the recognition of many different partners with a high specificity [21]. However, the promiscuity of IDP(R)s does not come without a cost with a number of diseases being characterized by IDPs participating in aberrant interactions such as the aggregation of α-synuclein into cytotoxic oligomers in Parkinson's disease [13,32].

### 3.2. Folding-upon-binding

Upon interaction with a partner, the vast majority of IDP(R)s adopt a well-defined three-dimensional structure, which is highly relevant as their function is only exerted once bound to a partner [33]. An IDP(R) transitions from a freely bound state, exhibiting extensive conformational freedom, to a folded state, where there is a decrease in conformational freedom. Achieving a high degree of complementarity within this interaction is completely dependent upon thermodynamics. To account for the loss in entropy, the interaction must be driven by enthalpic contributions from the non-covalent interactions formed within the IDP(R) and between the IDP(R) and partner. If the entropic penalty is well compensated, then a high nanomolar affinity may be achieved. However, due to the formation of only basic secondary structure being induced in the majority of IDP(R)s, the enthalpic contributions are often insufficient to counteract the entropic penalty, and interactions involving folding-upon-binding largely take place with a low affinity [4].

Two mechanisms are used to describe the coupled binding and folding of IDP(R)s, both based upon a two-state kinetic model. Conformational selection entails proteins binding to their partners and the intrinsic plasticity allowing a process of sampling structures that are complementary to the binding site [13]. The induced-fit model stems from the IDP(R) making non-specific contacts with the binding partner, inducing the disordered region to fold into the correct structure and form more specific interactions at the binding interface [13]. Typically, the interactions between the IDP(R)s and their target begin with hydrophobic residues from the disordered protein docking into hydrophobic patches located on the surface of the folded protein.

p53 can be used to demonstrate the phenomenon of folding-upon-binding. A metadynamics simulation conducted by Zou *et al.* [33] studied the interaction between p53 and MDM2 at an atomic level and concluded that the interaction proceeds via an induced-fit pathway. Initial contacts consisted of p53 docking five hydrophobic residues into a hydrophobic groove of MDM2. p53 specifically recognizes a multitude of partners that bind to overlapping regions of its MoRFs [34]. Each binding process involving MoRFs may be modulated by PTMs, further extending its list of partners [35].

The degree of induced folding can vary significantly. IDP(R)s may form extensive secondary or tertiary structures upon binding with only short segments taking an ordered state. The regions of the IDP that flank these ordered domains may remain disordered in complex [3].

## 4. Disordered protein domains

Molecular recognition of disordered proteins by their partners has commonly been assumed to involve disordered-to-ordered transitions [36]. This notion is incomplete as many IDPs retain a high level of their structural heterogeneity in the bound state and exert their function without the need for adopting a specific three-dimensional structure [37]. Moreover, in many dynamic complexes, the disordered regions are critical to delivering function or increasing the overall binding affinity for the complex. These complexes, where a significant degree of the conformational heterogeneity of an IDP is retained within the bound state, have been called ‘fuzzy complexes’ [38,39].

An IDP may bind to its partner and form complexes with varying degrees of disorder. The ‘fuzziness’ of a protein complex should be viewed as a broad spectrum based upon the relative entropy inherent to the complex.

The dynamic nature of fuzzy complexes results in fuzzy regions establishing transient interactions with the target, each coupled with a weak binding affinity. However, certain IDPs, for example, histone linker H1, have been identified to exhibit an extremely high-binding affinity with multiple partners [2]. This is due to the intrinsic properties of the IDP(R)'s primary sequence being rich in charged amino acid residues and giving the IDP(R) a polyelectrolyte nature [36].

### 4.1. Types of disordered complexes

The entropic penalty associated with IDP(R) binding may be minimized by the formation of fuzzy complexes. This ‘fuzziness’ may be separated into four classes according to static disorder and dynamic disorder: (i) the polymorphic model, which represents static disorder with alternative bound conformations serving distinct functions; (ii) clamp models, where IDRs link two or more globular domains; (iii) flanking complexes, where fuzzy regions neighbour bound and ordered domains and provide additional contacts, boosting the affinity; and (iv) random complexes, where transient interactions proceed from both partners with no induced secondary structure [1,38]. It is essential to note that these complexes are not mutually exclusive to one class. Disorder at the bound state is a continuum, and at any point in time a protein complex may exhibit character of more than one class.

An important point to note is that the interactions between IDP(R)s are not solely dictated by their reactive modules (SLiMs) but are mediated by the disorder and the intrinsic properties of the chain [40]. This review focuses on the contributions that are electrostatic in nature, manifesting as large stretches of amino acids with a large negative or positive charge density [19].

#### 4.1.1. Polymorphic model

The polymorphic model refers to static disorder, where an IDP(R) may adopt distinct, well-defined conformations in the bound state [38]. As a result of structural heterogeneity in the bound state, the selected electron density is missing from the solved structures, and the complex is classified as a disordered protein domain [38,41]. In the simplest case, a polymorphic model consists of two proteins in a bound state, adopting two unrelated three-dimensional conformations [38].

An example of a protein complex defined by the polymorphic model is the interaction between β-catenin (β-cat) and transcription factor-4 (Tcf4). β-cat is a multifunctional IDP which plays a crucial role in coordinating cell adhesion, transcriptional co-regulation and protein homeostasis [42,43]. The disordered catenin-binding domain of Tcf4 binds to β-cat in an extended conformation, while the acidic domain in the middle portion of Tcf4 adopts two distinct conformations by establishing alternative salt bridges between Asp16 of Tcf4 and Lys435 of β-cat [38,41]. In addition, the ability to form a dynamic complex between the two conformations limits the entropic penalty associated with binding, consequently making the overall binding process more thermodynamically favourable [38]. The plasticity of the interactions between β-cat and Tcf4 has recently been probed in further detail by Smith *et al.* [44], who showed that their association is a one-step process, while dissociation is two-step.

#### 4.1.2. Clamp model

The clamp model entails bound, globular domains connected by a disordered protein segment, called a ‘linker segment’ [39]. The ordered regions of the IDP serve as clamps and the disordered linker region may not interact with the target protein but is able to provide flexibility and adaptability in molecular interactions with a target [45].

Nuclear pore complexes (NPCs) are indispensable features of the eukaryotic cell, responsible for mediating nucleocytoplasmic transport. NPCs use phenylalanine-glycine-rich nucleoporins to control the transport of cargo proteins across the nuclear envelope [46]. These cargo proteins have integrated nuclear localization sequences (NLS) that are recognized by nuclear transport factors (NTFs). NTFs bind the cargo protein by the NLS and aid the transport of cargo through the NPC [47].

α-importin, a member of the karyopherin NTF family, binds the bipartite NLS of the molecular chaperone nucleoplasmin via a minor and major site, connected by a disordered linker region [48].

#### 4.1.3. Flanking model

An IDP(R) may bind to its partner through SLiMs, embedded within an environment of disorder [39]. The contact formed between an IDP and its target may be intermittent and electrostatic, polar or hydrophobic in nature. The remainder of the IDP exists as random coils and retains its conformational heterogeneity when flanking the interaction domain [39]. By retaining a degree of conformational freedom, the flanking region reduces the entropic penalty coupled with binding and makes this mode of interaction thermodynamically favourable. It has been widely reported that flanking regions modulate the nature of the interaction between an IDP and its target, contributing to binding affinity and specificity [40]. A review by Tompa & Fuxreiter [39] summarizes the increased binding affinity that flanking regions have on the complex. Moreover, Zor *et al.* [49] conducted a study measuring the dissociation constant (*k*_d_) while performing a series of amino acid deletions in a disordered flanking tail. A lower *k*_d_ value is observed when there is an increase in the number of deletions [39,49]. Equally, it has been observed that flanking regions may have no effect on the binding affinity of the complex [40]. Two modes of binding within the flanking model that this review will focus on are avidity and allovalency [21,40].

**Figure 2.**
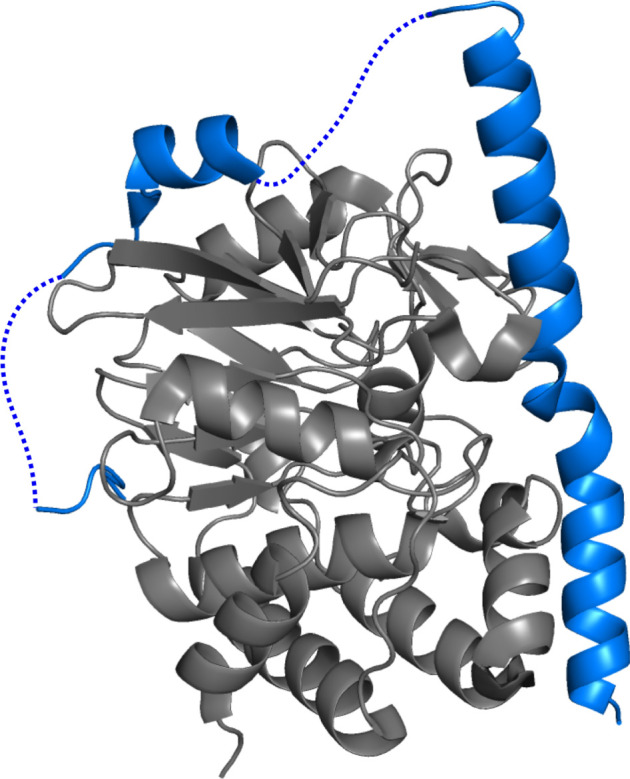
Avidity model. An X-ray structure of an IDP (blue)—inhibitor 2 (I2)—bound to rat protein phosphatase 1 catalytic subunit gamma isoform (PP1cgamma) (grey). The dotted lines on the IDP I2 indicate disordered regions not visible in the electron density, however upon binding to PP1cgamma two α-helical binding sites form. SLiMs located on these helices are complementary to two binding sites on PP1cgamma. PDB ID: 2O8A [50].

Avidity is a mechanism of binding which has been adapted from the model used to describe that of an antibody to an antigen [21]. Avidity entails two or more binding sites present on an IDP, linked by a disordered segment, that are complementary to two or more binding sites present on its partner [21]. A requisite of avidity is that the number of binding sites on both partners must be identical, and at no point in time may contacts interchange [21]. Regions of the IDP adjacent to the contacts remain disordered and flank the interaction domain (figure 2). An advantage of this binding mechanism is that once the initial contact is made, a cooperative effect takes place, facilitating further interactions [21,51]. Moreover, the increased local concentration from the first binding event, combined with the lowered entropic penalty when binding only one IDP results in a greater binding affinity and the interaction is more thermodynamically favourable [51]. Olsen *et al.* [21] modelled this kinetically by labelling the first interaction as second order, and the pseudo-intramolecular interaction as first order, with the rate of interaction being governed by the two rate constants and the effective concentration.

**Figure 3.**
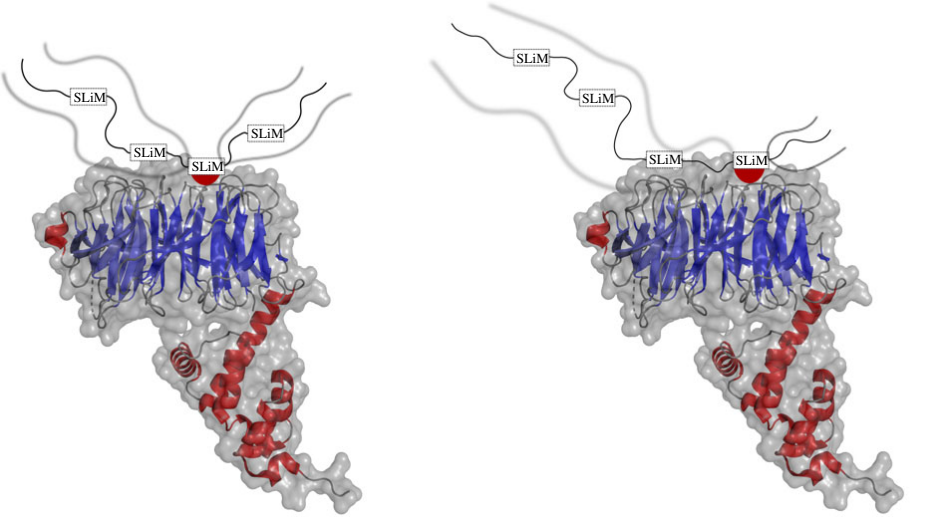
Allovalency model. An X-ray structure of the SCF E3 ubiquitin ligase components Cdc4 (blue) and Skp1 (red) with the disordered N-terminal region of the yeast cyclin-dependent kinase (CDK) inhibitor Sic1 (cartoon). Multiple phosphorylation sites are positioned in tandem on Sic1 and labelled as SLiMs. Each SLiM is complementary to a binding site on Cdc4 [52]. The dissociation of interaction at one site enables a new interaction at another site. The disordered regions remain flanking as indicated by the blurred lines. PDB ID: 3V7D [52].

Allovalency models a system where there are multiple binding sites positioned in tandem on an IDP that are complementary to a single binding site on its partner (figure 3) [21]. At any point in time, the IDP may only make one contact with its target. As a result, the competition between residues on an IDP for the binding site on its target is increased, giving rise to a larger local concentration. At any given moment, an interaction may dissociate, and due to the increased local concentration of IDP binding sites, the probability of another interaction being established increases. Modelled by Locasale [53], the probability of an IDP escaping its target decreases exponentially as a function of the number of binding sites. Furthermore, the increased local concentration has a net effect on increasing the overall binding affinity of the complex.

#### 4.1.4. Random model

The random fuzzy model describes a situation where both the IDP(R) and its target have (*n*) number of interaction sites, where interaction between each site is not restricted by any specificity [21]. Similar to allovalency, the probability of establishing an interaction following the dissociation of a previous interaction is increased by the larger local concentration. Random fuzziness represents the extreme case of disorder where there is little to no secondary structure induced upon the interaction between an IDP and partner [21,39]. The degree of conformation freedom present in the bound state, therefore, makes the characterization of these interaction domains extremely difficult [21]. A number of random model fuzzy complexes have emerged over the past five years involving highly charged IDP(R)s behaving as polyelectrolytes.

Polyelectrolyte behaviour is an inherent property of some IDPs due to a high charge density of amino acids. Recent advances in structural biology have probed the interactions of numerous nuclear proteins, such as histone linkers and their chaperones, both of which display large polyelectrolyte characters [2,54,55].

### 4.2. Disordered domains involving histone linker, H1

Histone linker H1 is a highly dynamic nuclear IDP involved in the remodelling of chromatin [56,57]. H1 consists of three domains: an unstructured N-terminal domain; a central winged-helix globular domain; and an acidic, disordered C-terminal domain (CTD) [54,57]. The basic CTD possesses a net charge of +53, exhibiting polyelectrolyte behaviour [2].

Turner *et al.* [36] investigated the complex formation of H1 and DNA and probed the dependence of the basic CTD on the interaction, along with the degree of secondary structure induced during the binding event. H1 remains disordered when free in solution and when bound to DNA. Two complexes were studied at physiological ionic strength and pH: a 1.86 : 1 molar ratio complex of H1 bound to a 36 bp fragment of DNA; and a 0.996 : 1 complex, consisting of H1 bound to a 20 bp fragment of DNA [36]. Using isothermal titration calorimetry, a relatively high-binding affinity was determined for both complexes (*k*_d_ 1.86 : 1, 36 bp-H1 = 292 nM, 0.996 : 1, 20 bp-H1 = 101 nM) [36]. Analysis of circular dichroism (CD) spectra also revealed that there was no indication of an induced secondary structure during the binding event, and so H1 remains disordered when interacting with DNA [36]. The idea of an IDP being able to form a high-affinity complex with a complete lack of specificity is one that defies the structure–function paradigm and sometimes meets with controversy.

In recent advances, a study by Borgia *et al.* [2] investigated an ‘ultra-high’ affinity, disordered complex between H1 and its nuclear chaperone, prothymosin-α (ProT*α*). This has provided a framework for studying the polyelectrolyte behaviour of IDPs and has influenced many follow-up studies, probing the physical concepts that govern the interaction between H1 and ProTα. ProTα is a negatively charged (−44), highly dynamic IDP that increases the mobility of H1 within the nucleus and moderates its chromatin condensing function [58]. The complex formed between the two IDPs has shown to display very little secondary structure when observed by nuclear magnetic resonance (NMR) and CD spectroscopy [2]. In both IDPs, there is a lack of hydrophobic residues and their interaction cannot induce the formation of a hydrophobic core, which is necessary to drive protein folding. The polyelectrolyte behaviour of the two IDPs results in persistent and sporadic electrostatic interactions, giving rise to a highly dynamic complex [2,55,59].

Despite the lack of specificity encoded at the binding interface, the complex formed between both IDPs has a very high affinity. Single-molecule Förster resonance energy transfer (sm-FRET) was used to probe the binding between the two IDPs and indicated a binding affinity ranging from picomolar to nanomolar affinities at physiological ionic strength (165–200 mM) [2,55,59,60]. The ability for H1 complexes to form with such high affinity may eliminate competition in densely populated regions, such as the nucleus.

**Figure 4.**
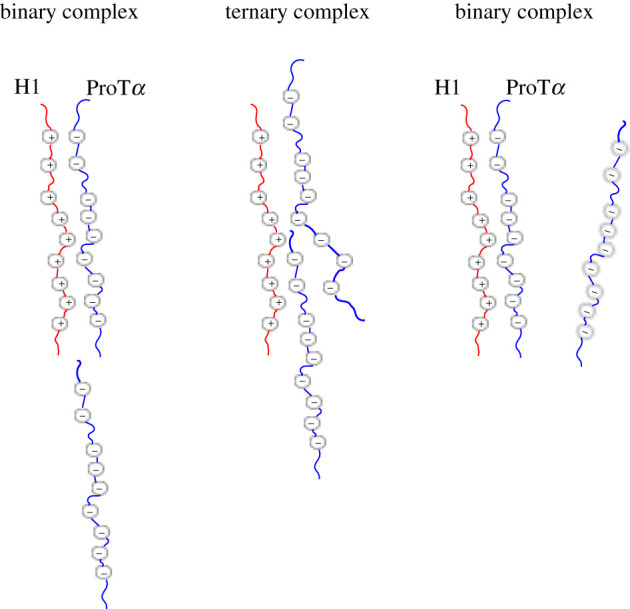
Competitive substitution model between H1 and ProTα. Histone linker H1 (red) is predominantly positively charged, while its nuclear chaperone ProTα (blue) is predominantly negatively charged. The proteins interact via electrostatics to form a binary complex. A second ProTα chain may attach to a region of H1 within the binary complex, forming a transient ternary complex, and compete with the initial ProTα chain for electrostatic interaction with H1.

The binding kinetics of the H1–ProTα complex was investigated by sm-FRET involving the addition of H1 to a fixed concentration of immobilized ProTα [59]. The protein-concentration-dependent dissociation rates obtained for the H1–ProTα complex deviated from a binary complex dissociation rate and suggested the existence of transient ternary complexes [55,59]. To gain insight into the molecular mechanisms involved with the formation of the ternary complex and rapid interconversion between bound and unbound ProTα, Sottini *et al.* [59] used coarse-grained molecular dynamics (MD) simulations. These simulations illustrated that the pronounced disorder in the binary complex facilitates the formation of the transient ternary complex (ProTα–H1–ProTα) [55,59]. Once the ternary complex is formed, either ProTα molecule may dissociate with equal probability [59]. This process has been called ‘competitive substitution’, and describes the action of ProTα interacting with the charged CTD of H1 while in a binary complex (H1–ProTα), resulting in the displacement of the original ProTα (figure 4) [55,59,61].

The competitive substitution model offers a description of the molecular mechanism involving the chromatin condensing function of H1. The complex formed between H1 and the nucleosome is completely disordered [36,55]. The fluctuations in the structure of H1 allow for ProTα to interact with the disordered CTD of H1 and compete with the electrostatic interactions between H1 and DNA [55]. Due to the high-affinity interaction formed between H1 and ProT*α*, ProTα is able to outcompete the nucleosome and remove H1 by competitive substitution [55]. This ability to form a ternary complex between ProTα-H1–DNA accelerates the dissociation of H1 bound to the nucleosome and allows ProTα to modulate the function of H1, acting as its chaperone.

### 4.3. Highly dynamic E-cadherin tail and β-cat interaction

Recent work by Wiggers *et al.* [62] identified a fuzzy complex, where segments of E-cadherin (E-cad) diffuse dynamically with high affinity over a large surface area of β-cat, in a manner distinct to the interactions observed between β-cat and Tcf4 [42–44,62]. β-cat is a cytoplasmic multifunctional repeat protein consisting of three domains: a central region domain, consisting of 12 imperfect armadillo domains which serve as the major interaction domain; a disordered N-terminal domain and a disordered CTD [62–65]. β-cat is a key component in moderating cadherin-based cell adhesions and was first identified in association with epithelial cell adhesion molecule E-cad [62,63]. Cadherins comprise a large family of transmembrane glycoproteins that mediate epithelial cell behaviour [65]. E-cad specifically mediates cell adhesion by linking actin filaments of adjacent epithelial cells by binding to the cytoplasmic tail of β-cat which, in turn, establishes contact to actin-associated protein, α-catenin [62]. X-ray crystallography data shows that the E-cad tail wraps around the central domain of β-cat during a 1 : 1 interaction; however, roughly half of the electron density is missing from the data suggesting that the E-cad–β-cat is highly dynamic [62,63].

Wiggers *et al.* probed the interactions between E-cad and β-cat using sm-FRET techniques to establish appropriate parameters used to define a coarse-grained molecular dynamics simulation. Initially, E-cad was isolated and screened free in solution and then in KCl [62]. E-cad exists in an extended structure in solution due to the electrostatic repulsions within its sequence (−22 charge) [62].

The 1 : 1 complex formed between E-cad and β-cat was monitored by nanosecond FCS (nsFCS) and sm-FRET studies to determine a relative binding affinity, dynamics and to define parameters used for CG MD simulations. The binding affinity of the complex was determined to be in the range of 4 ± 2 nM for all constructs, providing yet further evidence for high-affinity fuzzy complexes [62]. The information regarding the dynamics of the 1 : 1 complex was used to define a CG MD simulation which describes the strength of intermolecular contacts [62]. These revised parameters account for the strength of the intermolecular contacts and categorized amino acids based on being polar, charged or hydrophobic. The MD simulations indicated that the most abundant interactions occurred between charged and hydrophobic amino acid residues, deviating from our notions of fuzzy interactions being mediated by charge–charge interactions between the amino acids of the individual IDPs observed between H1 and ProTα [55,59,61,62]. Interestingly, the C-segment which appears to be resolved and static in the X-ray structure appeared to be dynamic, corroborating with data obtained in RASP dynamics methods and nsFCS [62]. The core binding region of E-cad also displayed a binding affinity of approximately 1 µM, implying that the many weak and non-specific contacts are crucial for boosting the overall binding affinity of the complex [62]. Importantly, the results from the MD simulation were matched with the experimentally obtained data, where a total of 91% of the surface area of β-cat was explored by E-cad [62].

### 4.4. Predicting the propensity of IDPs to undergo fuzzy interactions

Recent developments by Miskei *et al.* [31] established a novel scoring algorithm (FuzPred) to predict the propensity of an IDP to interact via a fuzzy complex mechanism without any knowledge of a binding partner. The local sequence is subject to an algorithm where individual amino acid residues are scored based on their propensity of undergoing disorder-to-disorder, disorder-to-order and context-dependent binding modes. This novel approach of predicting the mode of interaction for IDPs from their local sequence composition is of great importance for developing our understanding of the interactions associated with IDPs [31]. This approach shows huge potential as it has further developed to predict the propensity of amino acid residues to undergo aggregation within the droplet state for IDPs associated with pathological conditions, such as Parkinson's disease and Alzheimer's disease [16].

### 4.5. Disorder in protein structure prediction

There has been growing interest in the application of machine learning to the problem of protein folding in structural biology. The most successful implementation of this has been the AlphaFold project as demonstrated clearly in the 14th Critical Assessment of Structure Prediction (CASP14) [66,67]. AlphaFold is capable of generating accurate (up to a median backbone accuracy of 0.96 Å RMSD_95_) protein models by using a combination of evolutionary, geometric and physical constraints, and is already proving useful in providing search models for molecular replacement and in the interpretation of cryo-EM data [67,68]. AlphaFold has, with support from the European Bioinformatics Institute (EBI), been used to produce structures for 98.5% of proteins in the human proteome [69]. Of the total residues predicted as part of this effort 58% have been predicted confidently; this is defined by the authors as having a pLDDT (per residue confidence metric) greater than 70 and is reflective of an accurate backbone prediction. The remaining 42% of the residues, however, are in large part thought to reflect those residues that exist in disordered regions [69]. This is supported by estimates from the D^2^P^2^ database that 37–50% of all residues in the human proteome are disordered [70].

The bulk of structures deposited in the PDB are from X-ray crystallography (approx. 88% at the time of writing). An intrinsic limitation of this technique is its difficulty in handling disordered regions and flexibility, and so these regions are often removed from the expression construct to facilitate crystallization. It has been quite illuminating to see so much disorder in the full-length structural predictions output by AlphaFold—regions that have historically been overlooked—and these outputs will further drive interest in the field of IDPs. It should also be noted the success with which AlphaFold has identified disordered residues, at a level comparable to that seen among the best performers in the first Critical Assessment of Protein Intrinsic Disorder Prediction (CAID) [71]. We also hope that further innovations in machine learning with respect to protein structure (and unstructure) may shed further light on IDP behaviour and on the importance of fuzzy complexes in molecular biology.

## 5. Conclusion

Our understanding of protein structure and function is becoming increasingly challenged by the identification of disordered protein domains. The protein structure–function paradigm indicates that the function of a protein is encoded within complementary binding interfaces. IDP(R)s reshape this notion by their ability to function with an inherent lack of structure. Furthermore, the ability of IDP(R)s to interact promiscuously with multiple partners deconstructs the belief that function is solely dependent on a well-defined three-dimensional structure. Even the idea that IDP(R)s must adopt a fold when bound to a partner has been dismantled by the number of high-affinity random fuzzy complexes. A shift in our notions of the protein structure–function paradigm must account for the activity of IDP(R)s and how the amino acid sequence may encode interactions through MoRFs and SLiMs.

While the number of identified fuzzy complexes is increasing, there remains a lack of understanding of the kinetics and thermodynamics governing these interactions. For example, the linker histone H1 has been shown to behave as a polyelectrolyte and form ternary complexes during its interaction with ProTα. However, we do not know if there is a possibility of forming higher-order oligomers *in vivo,* and what effect this may have on the kinetics. The interaction between H1 and ProTα is mediated through the basic CTD of H1, but how does H1's charged globular domain interact with ProTα? To investigate these principles and gain a deeper understanding of how H1 operates within the nucleus, the amino acid sequence should be investigated with protein engineering—specifically, how the charged contacts and the charge positioning may affect the overall affinity of the complex. This information would contribute to a deeper understanding of disorder and allow us to comprehend how specificity may be achieved when there is an inherent lack of structure.

It should also be noted, however, that in many studies of IDP(R)s and the behaviour of their binding modules—including in the case studies described here—the constructs used are often ‘cut to the quick’ (i.e. the disordered regions are frequently taken outside their full protein context). While information gained from these studies is invaluable, there should be an awareness that IDP(R)s studied outside of their native, full-length polypeptide environment may behave differently, and as such where possible it should be encouraged that studies use the full-length proteins.

## Data accessibility

This article has no additional data.
